# Livestock 2.0 – genome editing for fitter, healthier, and more productive farmed animals

**DOI:** 10.1186/s13059-018-1583-1

**Published:** 2018-11-26

**Authors:** Christine Tait-Burkard, Andrea Doeschl-Wilson, Mike J. McGrew, Alan L. Archibald, Helen M. Sang, Ross D. Houston, C. Bruce Whitelaw, Mick Watson

**Affiliations:** The Roslin Institute and R(D)SVS, University of Edinburgh, Easter Bush, Midlothian, EH25 9RG UK

## Abstract

The human population is growing, and as a result we need to produce more food whilst reducing the impact of farming on the environment. Selective breeding and genomic selection have had a transformational impact on livestock productivity, and now transgenic and genome-editing technologies offer exciting opportunities for the production of fitter, healthier and more-productive livestock. Here, we review recent progress in the application of genome editing to farmed animal species and discuss the potential impact on our ability to produce food.

## Introduction

There are an estimated 7.6 billion humans on the planet, yet an estimated one in nine of us (821 million people) do not have access to sufficient food to lead a normal, active life [1]. Despite the problems we face feeding our species, the human population is set to grow, reaching 8.5 billion in 2030, 9.7 billion in 2050 and 11.2 billion in 2100 [2]. Clearly, if we struggle to feed 7.5 billion people currently, preparing to feed almost 4 billion more will be one of the biggest challenges facing our species**.**

The FAO (Food and Agriculture Organization of the United Nations) has published estimates that total agricultural output, from both crops and animals, needs to increase by 60% in order to meet demand. Importantly, this is being driven by a higher demand for animal protein, with some estimates that milk production will need to increase by 63%, and meat production by 76% [3]. This proportional increase in demand for animal products is largely driven by both population growth and increased affluence in low- and middle-income countries (LMICs). Terrestrial and aquatic animal production in these countries is heavily reliant on small-holder farmers, who collectively play a crucial role in global animal protein production. For example, of the 570 million farms worldwide, over 1 in 4 (150 million) have at least one milk-producing animal [4], and farms with fewer than 100 animals account for over 99.7% of global dairy production [5]. In LMICs, livestock accounts for over 60% of agricultural gross domestic product (GDP) [6], and farmed animals provide livelihoods for over 1 billion people globally [7]. While increasing reliance on plant-based diets is often raised as a potential solution to food insecurity and as part of the effort required to address climate change [8], omission of animal protein from human diets risks nutritional deficiencies and malnutrition [9]. There are also large geographical regions where livestock represent the most feasible land-use option, such as the dry lands that cover 60% of Sub-Saharan Africa [10].

In 2011, Sir John Beddington led a team of experts who examined the intricate links between global demand for food, energy and water. When placed within the context of climate change, he described the concurrent and rapid increase in demand for these commodities as “a perfect storm” [11]. The subsequent FORESIGHT report [12] identified six key pressures on global food production systems that already fail to feed the human population – global population increase, changes in consumer demand, changes in local and global governance, climate change, competition for key resources (e.g. clean water), and changes in the ethical stance of consumers. The goal of producing more food whilst using fewer resources is a major challenge for our species.

**Fig. 1:**
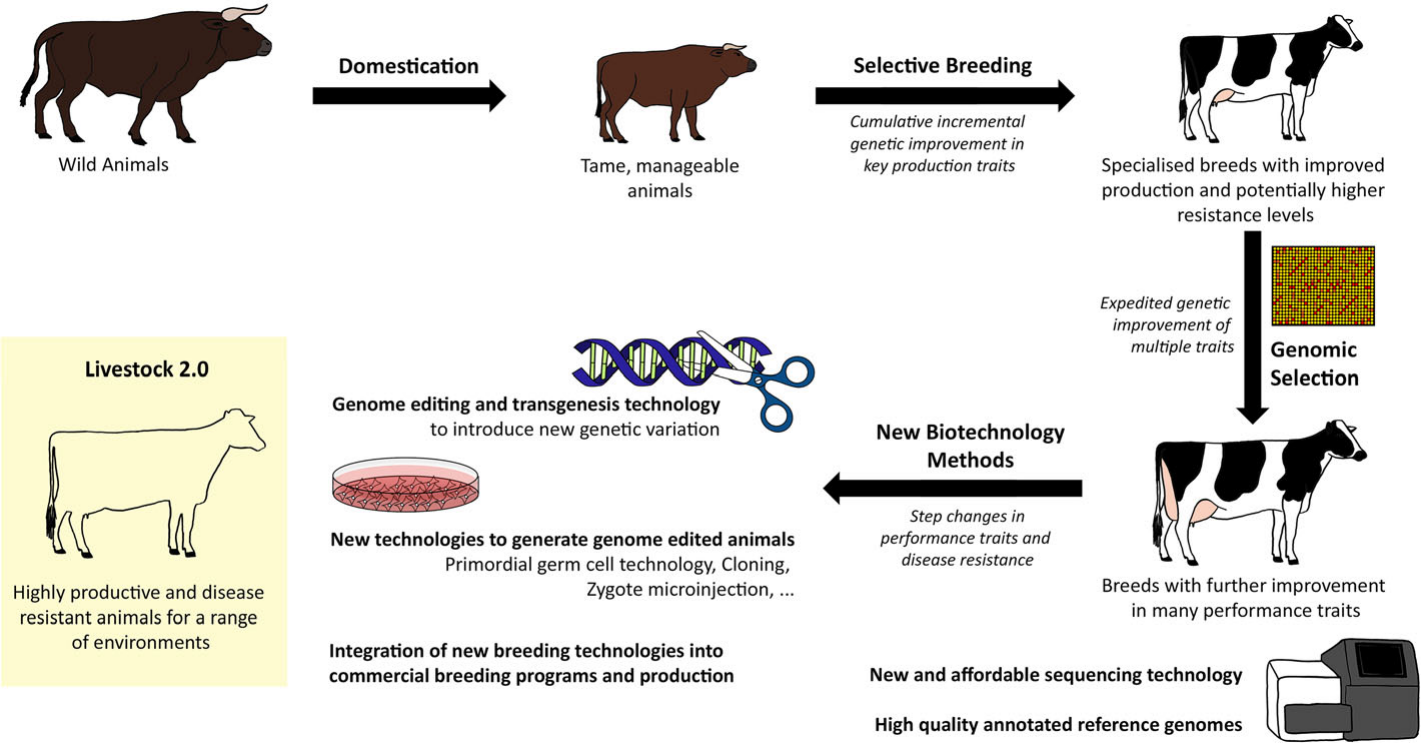
Pathways to ‘Livestock 2.0’. A brief summary of the developments in livestock breeding and what new technologies might offer to the industry. Selective breeding and genomic selection have already improved productivity and disease resistance in livestock significantly. Genome editing and transgenesis could facilitate step improvements through (i) rapidly increasing the frequency of favorable trait-associated alleles, (ii) introgression of favorable alleles from other breeds/species without linkage drag, and (iii) creation of de novo favorable alleles. A key challenge will be the identification of genome-editing targets, which will require a combination of high-quality annotated livestock genomes, well-powered genome-wide association studies, reverse-genetic screens (e.g. genome-wide CRISPR knock-out), and high-resolution knowledge of the biology of the target traits. CRISPR, clustered regularly interspaced short palindromic repeat

Here, we review the impact of genomics, transgenesis and genome editing on issues that influence farm-animal productivity, health and welfare, and on our ability to produce food, and go on to discuss the potential future impact of transgenic and genome-editing technologies (Fig. 1).

## The impact of genetic improvement on animal production

While many farmed animals have undergone the process of domestication for millennia, managed selective breeding programs have resulted in striking improvements in productivity. Genetic improvement has resulted in faster, cheaper, healthier, and more-efficient animal production, with reduced impact on the environment. For example, from the 1960s to 2005, selective breeding resulted in 50% larger litter sizes in pigs, an increase of lean pork meat of 37%, and a doubling of lean pork meat per kg of feed intake; in chickens, the days to acquire 2 kg of mass reduced from 100 days to 40, the percentage breast meat increased from 12 to 20%, the feed conversion ratio halved, eggs per year increased by 30% and eggs per tonne of feed increased by 80%; and finally, in cattle, milk production increased by 67% [13]. These transformative increases in food production represent incredible achievements in just a few decades, albeit the benefits were disproportionately seen in developed countries.

Pedigree-based breeding programmes for major livestock and aquaculture species now routinely incorporate genomic selection, which has been a revolutionary change for selective breeding and food production. Genomic selection [14, 15] involves the use of genome-wide genetic marker data to estimate genomic breeding values (GEBVs) of individuals by means of a genomic prediction equation. This genomic prediction equation is calculated using a ‘training’ or ‘reference’ population where animals have both genotypes and phenotypes, and is then applied to selection candidates, which often have marker genotype information only. The rates of genetic gain have been estimated to lie between 20 and 30% in cattle, pigs, chickens and salmon [16].

Genomic improvements have been accelerated by community-driven pre-competitive research in animal genomics and functional genomics. The major farm-animal genomes have been sequenced [17–19], with efforts under way to functionally annotate these genomes to the same standard as the human genome [20–22]. Some farm-animal genomes now represent the most contiguous complex genomes ever sequenced [23, 24]. Built on these efforts, genomic tools [25–30] and new and cheaper sequencing technologies [31, 32] have been, or will be, major contributors to modern animal breeding and the improved productivity of farmed animals.

Selective breeding is constrained by the standing genetic variation in the species or population of interest, and new variants arising through de novo mutations. Transgenic and genome-editing technologies offer new opportunities for genetic improvement by creating novel beneficial alleles or introducing known desirable alleles from other breeds or species, without the consequences of the linkage drag associated with traditional introgression. Below, we summarize some of the applications of both genetic modification and genome editing to farm-animal productivity and health.

## Examples of genetic modification

Genetic modification of farmed animals to increase the efficiency of food production, increase animal health and welfare, yet reduce the environmental footprint, has been a goal for many decades (Table 1). Early work focused on attempts to increase growth. Muscle development and body mass are controlled at a high level through the pituitary gland and liver, through the growth hormone–insulin-like growth factor axis (GH–GF axis) [33]. Growth hormone (GH) is released by the pituitary gland and stimulates the expression of insulin-like growth factor 1 (IGF1) in all tissues, including muscle. The predominant source of systemic IGF1 is the liver, and both muscle- and liver-derived IGF1 have been found to stimulate muscle hypertrophy. IGF2, a sister molecule of IGF1, has key roles in myogenesis [33], and mutations in a regulatory region of the *IGF2* gene are associated with an increased level of muscle growth in pigs [34]. Pursel and colleagues [35] successfully introduced genes encoding two growth-related hormones (GH and IGF-1) into pigs by DNA microinjection into zygotes. Two lines of pigs expressing the transgene encoding GH gained mass 11.1 and 13.7% faster than control pigs, and were demonstrated to have 18% more efficient feed conversion. The mechanism appeared to operate through resource diversion, with lower levels of subcutaneous fat, and increased development of muscle, skin and bone [35]. A subsequent study [36] focused only on IGF-1, with transgenic pigs having significantly less fat and significantly more lean tissue (albeit with growth rates similar to those of control pigs). Although pigs from the latter study had no health issues, the GH transgenic pigs suffered increased lameness, lethargy and gastric ulcers and possessed a lower ability to respond effectively to stress [37]. These deleterious characteristics led to the cessation of this project.

**Table 1:** Examples of transgenesis for disease resilience and other production traits

Genetic modification/transgenesis			
Trait	Species	Transgene	Reference(s)
Increased growth	Pig	Growth hormone (GH) and insulin-like growth factor 1 (IGF-1)	[35–37]
	Salmon	GH (Chinook salmon), promoter (Ocean pout)	[38]
Larger ratio of n-3 to n-6 fatty acids	Pig	Fat-1 (*Caenorhabditis elegans*)	[39–43]
Reduction of the environmental impact through phosphorous & nitrogen release reduction	Pig	Phytase (*Escherichia coli*)	[44]
		Phytase (*E. coli*), xylanase (*Aspergillus niger*), β-glucanase (*Bacillus lichenformis*)	[45]
Avian influenza resilience	Chicken	shRNA decoy (synthetic)	[48]
Mastitis resilience	Goat	Lysozyme (human)	[50]
	Cow	Lysostaphin (*Staphylococcus simulans*)	[52]
PRRSV resilience	Pig	Histone deacetylase HDAC6	[67, 94]

Abbreviations: *GH* growth hormone, *PRRSV* porcine reproductive and respiratory syndrome virus, *sh* short hairpin

A similar approach was taken in farmed salmon, which were genetically modified to produce a rapid-growth phenotype. The AquAdvantage salmon strain (AquaBounty Technologies Inc., MA, USA) shows improved growth relative to wild-type salmon (in specialized onshore production systems) owing to the integration of a growth hormone gene from a Chinook salmon (*Oncorhynchus tshawytscha)* together with a promoter from ocean pout (*Macrozoarces americanus*), a cold-water ray-finned fish, to drive increased expression of growth hormone. A landmark in the field of genetically modified (GM) food animals was the approval of this GM salmon strain as fit for human consumption by the US Food and Drug Administration and the Canadian Food Inspection Agency in 2017. The approval of this product for sale represents the first genetically engineered animal to be sold on the open market, and took approximately 25 years to reach this stage [38].

Transgenic technology, in some cases combined with genome editing, allows for the introduction of new properties to animal protein that could have potential benefits for the human diet. For example, Lai and colleagues generated cloned pigs that expressed the *fat-1* gene from the nematode *Caenorhabditis elegans* and that exhibited significantly reduced ratios of n-6 to n-3 fatty acids, which might have human health benefits [39]. Although some have questioned the value of such pigs [40], nevertheless others have also generated pigs carrying the *C. elegans fat-1* gene (which encodes an n-3 fatty acid desaturase) and have observed similar changes [41, 42], including Li and colleagues, who used ‘clustered regularly interspaced short palindromic repeats’ (CRISPR)–CRISPR-associated 9 (Cas9) gene-editing technology for the directed integration of the *fat-1* gene from *C. elegans* into the porcine *Rosa 26* locus [43].

Several groups have attempted to reduce the environmental impact of pig production through the addition of microbial genes into the pig genome [44, 45]. Dietary supplementation with phosphate and nitrogen is required to achieve optimal growth in many farm-animal species. Although phosphate, in the form of plant phytate, is present in their usual diet, farm animals are unable to digest it. The incompletely digested phosphorous and nitrogen are released into the environment through evaporation, manure and runoff and can disrupt local ecosystems. Golovan and colleagues [44] produced transgenic pigs that express an *Escherichia coli*-derived phytase gene, resulting in almost 100% digestion of dietary phytate, removing the need for phosphate supplementation. In a more recent effort, Zhang and collaborators [45] addressed the inefficient digestion of both phosphorous and nitrogen in pigs by generating transgenic pigs that not only expressed the microbial phytase enzyme but also xylanase and β-glucanase. This not only increased the growth rate of pigs by 23 and 24.4% in females and males, respectively, but also resulted in a reduction of fecal nitrogen and phosphorous by up to 45.8%.

Increased resistance to disease has been a goal of both selective breeding and genome engineering for many years. Avian influenza is an ongoing threat to poultry production world-wide, the economic impacts of major avian influenza outbreaks are very high [46] and the potential for development of human pandemic influenza infections is a continuing significant risk [47]. Avian influenza in production poultry can be controlled by vaccination or high biosecurity, but effective vaccines have yet to be developed, and high biosecurity cannot realistically be implemented by small-holder farmers. The potential of a novel alternative strategy – introduction of a transgene that would confer resistance to avian influenza – was tested by Lyall and colleagues [48]. Transgenic chickens were developed that expressed a short-hairpin RNA, based on the design described by Luo and colleagues [49]. This RNA molecule was designed to act as a decoy that blocks avian influenza virus polymerase, consequently inhibiting viral propagation. These transgenic birds were challenged with highly pathogenic avian influenza virus, and, although the birds succumbed to the initial challenge, transmission of the infection to transgenic and control birds was prevented.

Research has also focused on attempting to control mastitis using transgenic technology. Mastitis is one of the most common diseases of dairy cattle and has a huge negative impact on the industry, resulting in estimated annual losses of $2bn. The most common causative agent of persistent mastitis is *Staphylococcus aureus*, and resilience to this pathogen has a low natural heritability. Therefore, research has focused on transgenic strategies to create animals that express enzymes that inhibit the growth of mastitis-causing pathogens. Goats expressing human lysozyme have been shown to inhibit mastitis-causing bacterial growth [50, 51], whilst at the same time having little to no effect on the beneficial bacterium *Lactococcus lactis*, required for making dairy products such as cheese. Furthermore, transgenic cattle have been produced that express the antibiotic lysostaphin [introduced by somatic cell nuclear transfer (SCNT)] in their milk, which can prevent infection by *S. aureus* [52].

The above examples of transgenic animals generally predate the advent of genome-editing technologies. Genome editing by zinc-finger nucleases (ZFNs), transcription activator-like effector nucleases (TALENs), and CRISPR–Cas9 (and related enzymes) is known to be more accurate and efficient than transgenesis. Below we provide examples of early successes of the technology in farmed animals.

## Examples of genome editing

Compared with genetic modification, which relies on the incorporation of transgenes to enhance traits in livestock, genome editing offers an opportunity to make specific and precise changes to the genome of an animal to increase productivity and disease resistance. The myostatin gene (*MSTN*) is a common target for research into increased growth and muscle development. First identified in heavily muscled cattle and sheep breeds, such as Belgian Blue and Piedmontese cattle and the Texel sheep breed, it was found that an underactive gene for myostatin (also known as growth differentiation factor 8, GDF8) results in increased muscle growth. The underlying genetic variations are changes in the myostatin gene directly – an 11-bp deletion in the Belgian blue and a single-nucleotide polymorphism in the Piedmontese [53, 54]. Interestingly, the Texel breed encodes a regulatory mutation in the myostatin gene untranslated region (UTR), creating a target site enabling downregulation of the myostatin mRNA by two microRNAs [55]. Thus, the myostatin gene was an obvious early target for gene editing in farmed animal species as disruption of this single gene has significant effects on a trait of economic importance. To date, the farmed animals in which the myostatin gene has been edited include cattle [56], sheep [56, 57], goat [58] and Channel Catfish [59] (Table 2). The pig myostatin gene, however, has been the most frequently targeted [60–66] – perhaps as pork is the leading global source of meat by weight, or perhaps because of the lack of natural disruptive mutations detected in this gene to date. The first report by Ning Li and colleagues at the 10th World Congress on Genetics Applied to Livestock Production [67] reported problems in the pigs that were homozygous for a myostatin knockout, including the development of abnormal legs, an inability to stand and walk, and death. Similarly, mutations in myostatin have recently been associated with a recessive leg weakness syndrome in pigs [68]. Although Kang et al. [60] reported hypermuscling, increased lean, and reduced backfat in pigs with gene-edited knockouts of the myostatin gene, they also reported some health issues in the homozygous knockout pigs, and homozygous myostatin knockout Landrace piglets died only a few days after birth [65]. More recently, Wang et al. disrupted the myostatin gene in Erhualian pigs [61] and observed some double-muscle associated phenotypes. Although no health issues were reported, further characterization of the edited animals is required. Erhualian and Meishan pigs are two Chinese breeds known for high fat levels, and edited pigs with disrupted myostatin genes on these genetic backgrounds appear to fare better than those on leaner genetic backgrounds [61, 62].

**Table 2:** Examples of genome editing for disease resistance and other production traits

Genome editing			
Trait	Species	Genome-editing target	Reference(s)
Increased muscle growth (double-muscle phenotype)	Cow	Myostatin (GDF8)	[56]
	Sheep	Myostatin (GDF8)	[56, 57]
	Goat	Myostatin (GDF8)	[58]
	Channel Catfish	Myostatin (GDF8)	[59]
	Pig	Myostatin (GDF8)	[60–66]
Hornlessness (Polled)	Cow	Pc POLLED	[74]
Boretaint (Hormone release during sexual maturity leading to undesired meat taste)	Pig	KISS1R	[75]
Sterility	Salmon	Dead end protein (dnd)	[78]
Sterility/surrogate hosts	Pig	Nanos2	[79]
	Chicken	DDX4 (Vasa)	[80]
PRRSV resistance	Pig	CD163	[90–93]
ASFV resilience	Pig	RELA	[95, 96]
*Mannheimia* (*Pasteurella*) *haemolytica* resilience	Cow	CD18	[97]
Bovine tuberculosis resilience	Cow	NRAMP1	[99]
Xenotransplantation (removal of endogenous retroviruses)	Pig	Porcine endogenous retrovirus genes	[106, 107]

Abbreviations: *ASFV* African swine fever virus, *GDF* growth and differentiation factor, *PRRSV* porcine reproductive and respiratory syndrome virus

Beyond growth phenotypes, there has been a focus on more-efficient farming practices and animal and human welfare. Physical dehorning has many benefits to cattle, their handlers and the farming industry, including reduced risk of injury, reduced competition for feeding-trough space, and fewer aggressive behaviors [69]. It has been estimated that 80% of dairy farmers in Italy [70] and 93% [71] of dairy farmers in the USA practice routine dehorning of dairy cattle. Despite the benefits, dehorning of dairy cattle represents an animal-welfare concern, owing to the pain caused and potential for injury. Naturally hornless cattle (termed ‘polled’) do exist and are far more prevalent in beef cattle than in dairy. The genetic cause of polled cattle has been the subject of intense genetic research, resulting in the suggestion that one of two alleles must be causal [72, 73]. Carlson and colleagues [74] used TALEN to introduce the Pc POLLED allele into the genome of bovine embryo fibroblasts from four lines of cattle. These were cloned using somatic-cell transfer, resulting in full-term pregnancies for three of the four lines. Five live calves were produced; however, only two were viable and went on to survive to day 60 (at the time of publication). All five calves were determined to have a likely polled phenotype at birth, and the two surviving calves were confirmed to be polled. Not only does this confirm the causality of the Pc POLLED allele, but it also represents a potential approach for reducing physical dehorning in dairy cattle without a loss of productivity.

Surgical castration of pigs is a common practice in pork production to reduce aggressive behaviour and to avoid the accumulation of androstenone and skatole, which leads to the boar taint taste and odor of non-castrated male pork. Sonstegard and colleagues generated pigs with a knock-out of the *KISS1R* gene, encoding a receptor responsible for the onset of puberty in vertebrates and involved in the regulation of gonadotropin-releasing hormone [75]. The knock-out pigs showed a lack of testicular development but reacted to hormone treatment, which increased testicular size. However, it remains to be tested whether the animals can become fertile and whether growth properties are affected. Genome-wide association studies (GWAS) further highlight that the boar taint components and testicular trait regions have pleiotropic effects, which might impact the applicability of genetic interventions for this trait [76, 77].

Sterility has also been a focus in farmed Atlantic salmon, with a view to avoid escapees interbreeding with wild stocks. Genome-editing approaches have also been successfully applied [78], with the initial target being the dead end protein (encoded by the *dnd* gene) in order to induce sterility.

Research has also focused on methods to integrate genome-editing technologies into existing genomic-selection strategies. For example, a major barrier to the adoption of genomic selection in some areas has been the reliance on techniques such as artificial insemination of high-value germplasm, which relies on skills and infrastructure that are not accessible in all parts of the world. One solution is to generate sterile host animals that can be used to distribute transplanted high-value germplasm. Specific gene ablation of loci important for germ-cell development can generate animals that lack endogenous germ cells in homozygous individuals. Animals can then be distributed that will carry high-quality transplanted germplasm to geographic regions that are not serviced by the infrastructure needed for cryopreserved semen distribution. Both sterile pigs and sterile chickens have been produced using genome-editing technologies [79, 80]. Sterile surrogate hosts for poultry are especially valuable as cryopreservation methods in poultry are lacking. All poultry flocks are kept as breeding populations as it is impossible to freeze the chicken egg, and cryopreservation of chicken semen is inefficient and breed specific [81]. The early diploid germ cells of poultry can be cryopreserved and form functional gametes when transplanted into surrogate host chickens [82]. When transplanted into sterile surrogate chickens, it is now possible to reconstitute pure poultry flocks from frozen material [83, 84].

Finally, as with transgenesis, many groups focus their research on the potential of genome editing for control of infectious diseases (Table 2). Here there are clear opportunities, especially in cases where conventional control options have shown limited success. The development of pigs resistant to porcine reproductive and respiratory syndrome virus (PRRSV) exemplifies this strategy. PRRS is arguably the most important infectious disease problem for the pig industry worldwide. The losses from PRRS are estimated at $2.5 billion per annum in the USA and Europe alone. Quantitative genetics studies have identified substantial genetic variation in the resistance and tolerance of pigs to PRRS [85, 86], with a single locus on pig chromosome 4 (*GBP5*, encoding guanylate-binding protein 5) explaining 15% of the total genetic variation in viral load and 11% of genetic variation for growth rate in pigs infected with PRRSV [87, 88]. Although these results could offer promising opportunities for mitigating PRRS through genomic selection, predicting the impact of genomic selection on PRRS prevalence is difficult as the role of the *GBP5* locus in PRRS transmission is currently not known. In vitro research has shown that the macrophage surface protein CD163 and specifically the scavenger receptor cysteine-rich domain 5 (SRCR5) of the CD163 protein mediate entry of PRRSV into the host cell [89]. Based on this information, genome-edited pigs could be generated with a disruption to the *CD163* gene, giving rise to resistance to PRRSV infection. Whitsworth and colleagues knocked-out the *CD163* gene completely by the introduction of a premature stop codon by means of non-homologous end-joining events in exon 7 [90, 91]. A subtler approach by Burkard et al. removed only the SRCR5-encoding genome section, a deletion of exon 7, thus maintaining the expression and biological function of the *CD163* gene [92, 93]. Both approaches resulted in resistance to PRRSV infection [90–93], in contrast to the partial resistance conferred by the *GBP5* genotype in existing pig populations. Transgenic strategies to enhance resistance to PRRSV infection have also been attempted, including overexpression of histone deacetylase 6 (HDAC6), with the resulting transgenic pigs showing lower viral load and longer survival [67, 94]. However, such studies do not deliver the complete resistance observed in the pigs in which the endogenous *CD163* gene has been edited.

African swine fever (ASF) is another hugely important disease of pigs. Caused by African swine fever virus (ASFV), ASF is a disease endemic to huge swathes of sub-Saharan Africa, which has recently been introduced to Eastern Europe, from where it is rapidly spreading to Western Europe as well as China. Native *suid* hosts, including the warthog, are resilient to the infection, whereas domestic pigs develop a lethal haemorrhagic fever mainly caused by a cytokine storm in the host. Variation in the *RELA* gene between resilient and susceptible *suidae* has been postulated to underlie this phenotype [95]. RELA is a component of the NF-κB transcription factor, known to play a role in stress and immune responses. Using a ZFN, researchers were able to convert the domestic pig protein sequence for RELA to that of the warthog [96] – however, data to show resilience to ASFV have yet to be reported.

Genome editing offers the potential for control of several other diseases. *Mannheimia* (*Pasteurella*) *haemolytica* infection causes epizootic pneumonia (shipping fever), enzootic pneumonia and peritonitis in calves, lambs and sheep. *M. haemolytica* produces a leukotoxin that is cytotoxic and that binds to the uncleaved signal peptide of the CD18 protein on the surface of leukocytes. However, in other species that do suffer disease (e.g. mouse and human), the mature CD18 lacks the signal peptide. ZFNs have been used to introduce a single amino acid change in the cattle CD18 protein, and leukocytes from the resultant fetuses were resistant to *M. haemolytica* leukotoxin-induced cytotoxicity [97]. Bovine tuberculosis (bTb) is a potential zoonotic that has a huge and negative impact on productivity in cattle and buffalo. Polymorphisms in the *NRAMP1* gene in cattle have been associated with resilience to bTb [98]. Insertion of the resilient *NRAMP1* allele into cattle using CRISPR–Cas9 has been performed by Gao et al. [99]. Peripheral blood monocytes challenged with *Mycobacterium bovis* showed reduced pathogen growth, and an in vivo study using the edited animals reported a diminished interferon response.

The success of gene-edited animals in disease control will be influenced by many factors – for example, the proportion of gene-edited animals in the population and how these are distributed within and across farms. According to epidemiological theory, only a proportion of gene-edited animals would suffice to achieve herd immunity – that is, prevent disease from spreading within local populations [100]. Improved, disease-specific epidemiological models can help define the exact proportion of gene-edited animals needed for each species/disease, influenced by population structure, demographic characteristics, diverse environmental factors and management strategies influencing transmission dynamics, and the effectiveness of genome editing.

A common aspect of disease mitigation strategies is that of limited shelf-life. Genome editing shares the potential risk of vaccines in that its efficacy might be time limited owing to emergence of escape mutants [101]. For an RNA virus such as PRRSV with extremely high mutation rates [102], this seems a justified concern. Hence the question is not only “how many gene-edited pigs do we need to control disease?”, but also “how fast can these be realistically disseminated?”

It is important to differentiate between disease resistance, the ability of an animal to suppress the establishment and/or development of an infection, and disease resilience, where an infected host manages to maintain an acceptable level of productivity despite challenge pressure. For example, in the case of African swine fever, genome editing might primarily boost the tolerance of pigs to infection, rather than their resistance to becoming infected. Although genetic improvement of tolerance is considered to impose less risk for pathogen evolution towards higher virulence than genetic improvement of resistance, genetically tolerant individuals do not stop disease from spreading. In fact, the presence of genetically tolerant individuals that do not develop symptoms when infected, within a mixed population, might enhance disease incidence and prevalence.

Although not related to food production, a fascinating potential use of livestock is in the production of organs for human transplantation. Here also genome editing has a role. Xenotransplantation describes the process of transplanting an organ from one species into another and has become a hot topic of research owing to the lack of suitable human donors [103]. Pigs have been a natural focus of xenotransplantation research owing to their similarity in physiology and size – however, there are concerns over the risk of retroviral transmission from pig to human [104, 105]. Porcine endogenous retroviruses (PERVs) are retroviruses found within the genome of all pigs. As they are integrated into the genome, they exist in all tissues and organs and are passed on by inheritance. Genome editing is one possible avenue for removing or inactivating PERVs within pig genomes, thus making their organs suitable for xenotransplantation. Yang and colleagues [106] demonstrated this first, inactivating all 62 PERVs within the genome of a pig cell line (PK15) and reducing the levels of transmission to human cells by over 1000 fold. A follow-up study by Niu et al. [107] generated PERV-inactivated pigs through SCNT, having inactivated all the PERVs in a porcine primary cell line using CRISPR–Cas9. Genomic and transcriptomic analysis of the resulting pigs suggested 100% elimination of PERV activity.

## Discussion and future outlook

For many years genetics/genomics and selective breeding have had a transformative impact on livestock production and health, producing huge gains for the breeding industry, farmers and consumers. Underpinned by genomic tools and reference datasets, genomic selection has been (or is being) adopted worldwide to deliver consistent, predictable improvements in multiple species and farming systems. While selective breeding has resulted in successful incremental improvements in target traits, it typically relies on naturally occurring genetic variation within a population.

Transgenic and genome-editing technologies offer the opportunity for larger gains over a shorter time-period and can call on variation present in other populations and species, variation in non-domesticated species, and novel alleles designed to be beneficial. Resilience to ASFV is a potential example whereby an allele only present in the wild warthog population, which has co-evolved with the pathogen for many thousands of years, has been introduced into domesticated pigs by genome editing. Although we do not know the phenotype of the edited pigs, the concept of introducing beneficial alleles from a wild population into domesticated equivalents is sound. The allele conferring resistance to PRRSV introduced by Burkard and colleagues is an example of a ‘designer allele’ – the researchers knocked out a single exon of the CD163 gene, thought to be involved in interactions with the virus, and this simple edit appears to have produced resistant pigs that maintain normal CD163 functionality. To our knowledge, pigs lacking this exon have never been seen in any population, and therefore equivalent pigs would be impossible to produce by either artificial or natural selection. Elimination of this devastating disease of pigs could now be possible through the use of genome-edited pigs.

The older transgenic technologies have been applied to livestock since the 1990s, and there are many examples in this review – but why have so few engineered animals actually made it to market? For transgenic animals, the answer might come from the only success story, the AquAdvantage salmon. This product took 25 years to reach the market, with the first application for FDA approval occurring in 1995 [108]. Clearly, a more rapid approach to regulatory clearance is needed if more transgenic products are to hit the market and provide advantages to consumers, farmers, and breeders alike.

It is clear that precise, accurate genome-editing techniques are very different in nature to transgenesis. The legal regulatory paths for genome-edited animals have yet to be established, and all of the examples covered herein are at a very early stage. However, huge strides have been made, and in particular the PRRS-resistant pigs produced at Missouri and Roslin offer great potential to eradicate or minimize this devastating disease. Effectively removing PRRS from pig farms would benefit farmers, consumers, and the pigs themselves. Other examples are not far behind, and, if much-hoped-for progressive regulatory pathways are established, then the effects on livestock production could be huge.

The examples described above naturally involve single alleles of large effect that are amenable to genome editing. Beyond these simple examples, many traits of interest are complex – that is, they are governed by many alleles, each of small effect. To achieve significant impact from genome editing by harnessing existing genetic variation for a complex trait, one would need to edit multiple alleles simultaneously, and editing approaches would need to be routinely integrated within commercial breeding-programme operations. Simulations have shown that, even with complex traits, genome editing could have a role in livestock improvement, either by increasing the frequency of favorable alleles [109] or removing deleterious alleles [110] as part of a genomic-selection-driven breeding programme.

Assuming that the regulatory pathways can be defined, and considering that genome editing is precise and quick, there must now be a renewed focus on the identification of editing targets. In the examples above, the identification of the target genes has come from a wide variety of approaches encompassing genetics, genomics, large-scale CRISPR-based functional screens, host–pathogen interactions, virology, bacteriology and serendipity. Although the latter cannot be planned for, it is clear that all of the other approaches, within an integrated, co-ordinated international programme of research, have the potential to identify targets that can provide huge benefits to the livestock sector and will have a transformational impact on the ability of our species to produce sufficient food in an environmentally sustainable way.

## Abbreviations

ASF: African swine fever
ASFV: African swine fever virus
bTb: Bovine tuberculosis
CRISPR: Clustered regularly interspaced short palindromic repeat
FAO: Food and Agriculture Organization of the United Nations
GM: Genetically modified
LMIC: Lower- and middle-income country
PERV: Porcine endogenous retrovirus
PRRS: Porcine reproductive and respiratory syndrome
PRRSV: Porcine reproductive and respiratory syndrome virus
TALEN: Transcription activator-like effector nuclease
UTR: Untranslated region
ZFN: Zinc-finger nuclease
